# ^18^F‐fluorodeoxy‐glucose positron emission tomography pattern and prognostic predictors in patients with anti‐GABAB receptor encephalitis

**DOI:** 10.1111/cns.13767

**Published:** 2021-11-26

**Authors:** Xiao Liu, Tingting Yu, Xiaobin Zhao, Gongfei Li, Ruijuan Lv, Lin Ai, Qun Wang

**Affiliations:** ^1^ Department of Neurology Beijing Tiantan Hospital Capital Medical University Beijing China; ^2^ China National Clinical Research Center for Neurological Diseases Beijing China; ^3^ Department of Nuclear Medicine Beijing Tiantan Hospital Capital Medical University Beijing China; ^4^ Collaborative Innovation Center for Brain Disorders Beijing Institute of Brain Disorders Capital Medical University Beijing China

**Keywords:** autoimmune encephalitis, diagnosis, gamma‐aminobutyric‐acid B receptor, outcome, positron emission tomography

## Abstract

**Aims:**

To identify the metabolic pattern and prognostic predictors in anti‐gamma‐aminobutyric‐acid B (GABAB) receptor encephalitis using ^18^F‐fluorodeoxy‐glucose positron emission tomography (^18^F‐FDG‐PET).

**Methods:**

Twenty‐one patients diagnosed anti‐GABAB receptor encephalitis who underwent ^18^F‐FDG‐PET at first hospitalization were retrospectively reviewed. ^18^F‐FDG‐PET images were analyzed in comparison with controls. Further group comparisons of ^18^F‐FDG‐PET data were carried out between prognostic subgroups.

**Results:**

^18^F‐FDG‐PET was abnormal in 81% patients with anti‐GABAB receptor encephalitis and was more sensitive than MRI (81% vs. 42.9%, *p* = 0.025). Alter limbic lobe glucose metabolism (mostly hypermetabolism) was observed in 14 patients (66.7%), of whom 10 (10/14, 71.4%) demonstrated hypermetabolism in the medial temporal lobe (MTL). Group analysis also confirmed MTL hypermetabolism in association with relative frontal and parietal hypometabolism was a general metabolic pattern. After a median follow‐up of 33 months, the group comparisons revealed that patients with poor outcome demonstrated increased metabolism in the MTL compared to those with good outcome.

**Conclusion:**

^18^F‐FDG‐PET may be more sensitive than MRI in the early diagnosis of anti‐GABAB receptor encephalitis. MTL hypermetabolism was associated with relative frontal or parietal hypometabolism and may serve as a prognostic biomarker in anti‐GABAB receptor encephalitis.

## Graphical abstract

First, we revealed pronounced MTL hypermetabolism detected by semi‐quantitative brain ^18^F‐FDG‐PET in patients with anti‐GABAB receptor encephalitis compared to controls, and ^18^F‐FDG‐PET should be considered when patients with normal MRI in suspected anti‐GABAB receptor encephalitis; second, MTL hypermetabolism may be a sensitive diagnostic and prognostic biomarker for anti‐GABAB receptor encephalitis.

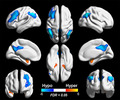

## 1 INTRODUCTION

Part of a group of severe but treatable neurological diseases, anti‐gamma‐aminobutyric‐acid B (GABAB) receptor encephalitis is a novel form of autoimmune encephalitis (AE) associated with antibody to GABAB receptor of cell surface.^1^, ^2^ The majority of patients with anti‐GABAB receptor encephalitis present with new‐onset seizures, cognitive deficits, and mental and behavioral disorders, all with or without the presence of underlying small cell lung cancer (SCLC).^3^ Early recognition of anti‐GABAB encephalitis is of vital importance because most patients respond well to timely treatment.^4^, ^5^, ^6^ Current diagnostic criteria depend highly on positive GABAB antibody in serum or cerebrospinal fluid (CSF), which can lead to false negatives or unavailable results, causing diagnostic difficulties and treatment delay.^7^ Thus, it is necessary to consider an additional novel biomarker in early diagnosis and prognostic evaluation for anti‐GABAB receptor in encephalitis patients. Although neuroimaging plays a key role in the routine evaluation of neurological diseases,^8^ it has received little attention for anti‐GABAB receptor encephalitis. Some prior studies have shown that magnetic resonance imaging (MRI) in these patients have mainly presented with hyperintensity signals in medial temporal lobe (MTL), but only 18%–50% patients have abnormal and specific results.^3^, ^9^, ^10^ ^18^F‐fluoro‐2‐deoxy‐d‐glucose positron emission tomography (^18^F‐FDG‐PET) is frequently used for whole‐body tumor screening, but recently, it has been reported to demonstrate MTL hypermetabolism in patients with anti‐GABAB receptor encephalitis, especially when MRI was negative.^11^ Nevertheless, only a limited number of isolated cases have suggested that ^18^F‐FDG‐PET might be useful for early diagnosis in subjects with anti‐GABAB receptor encephalitis, and there are no current data available to determine the sensitivity of brain ^18^F‐FDG‐PET in encephalitis associated with GABAB receptor antibody.^11^, ^12^, ^13^, ^14^ Moreover, the majority of previous studies of ^18^F‐FDG‐PET in anti‐GABAB receptor encephalitis have been restricted to qualitative characterization of FDG‐PET findings.^15^ In addition, to our knowledge, there is no systematically relevant study to evaluate the correlation between PET index and clinical prognosis in patients with anti‐GABAB receptor encephalitis. Overall, the metabolic pattern and prognostic role of ^18^F‐FDG‐PET in patients with anti‐GABAB receptor encephalitis are still not well described.

To address these unclear questions, we conducted a semi‐quantitative study reviewing the ^18^F‐FDG‐PET data of 21 patients with a definite diagnosis of anti‐GABAB receptor encephalitis. One aim of this study was to identify the ^18^F‐FDG‐PET pattern of anti‐GABAB receptor encephalitis, especially in those with unremarkable MRI changes. Further, this study also sought to find an imaging prognostic predictor by evaluating the association between metabolic signature and prognosis in anti‐GABAB receptor encephalitis.

## 2 METHODS

### 2.1 Study participants

The study was approved by the ethics committee of the Beijing Tiantan hospital that was affiliated to the Capital Medical University of the People's Republic of China. The study was conducted in accordance with the Declaration of Helsinki, and all patients and controls provided informed consent for the use of their medical records.

A total of 21 patients with GABAB receptor encephalitis were retrospectively reviewed between March 2015 and December 2020 at the Department of Neurology in the Beijing Tiantan Hospital of the Capital Medical University. All patients underwent MRI and ^18^F‐FDG‐PET at first hospitalization and fulfilled the included clinical diagnostic criteria based on representative clinical symptoms and the presence of purely positive GABAB receptor antibodies in the serum and cerebrospinal fluid (CSF). The neuroimaging data (MRI and PET) carried out in the acute stage of encephalitis after symptom onset were included. The demographic, clinical presentation, laboratory testing, and electroencephalograph (EEG) were collected by searching the electronic medical records.

For individual or group analysis of ^18^F‐FDG‐PET, the clinical cohort was matched for age and gender with 30 controls (19 males, 11 females; median age, 52 years; interquartile range [IQR], 43–56 years; range, 33–66 years) who had normal neurological assessment and functional metabolism from Beijing Tiantan Hospital of the Capital Medical University.

### 2.2 Antibody test

All patients underwent serum and CSF antibody detection, including classical paraneoplastic antibodies (Hu, Yo, Ri, Ma2, CV2, amphiphysin), N‐methyl‐D‐aspartate receptor (NMDAR), leucine‐rich glioma inactivated‐1 (LGI1), contactin‐associated protein‐2 (CASPR2), GABAB receptor, α‐amino‐3‐hydroxy‐5‐methyl‐4‐isoxazolepropionic acid receptor (AMPAR), and glutamic acid decarboxylase 65 (GAD65). Serum and CSF samples were tested for the presence of isolated GABAB receptor antibodies, using both cell‐based assays (Euroimmun, Lübeck, Germany) and immunohistochemical analyses in the neuroimmunology laboratory of the Peking Union Medical College Hospital.

### 2.3 MRI

All MRI examinations were acquired using a 3.0 Tesla Siemens Trio MRI scanner (Siemens Healthcare). The standard MRI protocols included T1‐weighted imaging [T1WI, repetition time (TR) = 2000ms, echo time (TE) = 20 ms, field of view (FOV) = 250 mm × 220 mm, matrix = 400 × 250], T2‐weighted imaging (T2WI, TR = 6,200 ms, TE = 90 ms, FOV = 240 mm × 220 mm, matrix = 3,850 × 385), fluid‐attenuated inversion recovery (FLAIR, TR = 11,000 ms, TE = 120 ms, FOV = 250 mm × 221 mm, matrix = 240 × 160), and diffusion‐weighted imaging (DWI, TR = 2,600 ms, TE = 60 ms, FOV = 230 mm × 230 mm, matrix = 140 × 130). MRI results were independently evaluated by two experienced neurologists and radiologists (Qun Wang, Lin Ai). In case of obvious discordance in their initial evaluations, an informed consensus statement was reached.

### 2.4 ^18^F‐FDG‐PET acquisition and statistical parametric mapping (SPM) analysis

^18^F‐FDG‐PET was performed according to previously published methods.^16^ PET images were acquired using a PET/CT scanner (Elite Discovery, GE HealthCare). All patients fasted for at least 6 h, and fasting blood glucose levels could not exceed 8 mmol/L. No patients received neuroleptic drugs before undergoing FDG‐PET. ^18^F‐FDG was intravenously injected at a dose of 3.7–5.0 MBq/kg within 1 min, and subsequent uptake required that patients be in a quiet resting status for 1 h prior to scanning in a dedicated room after ^18^F‐FDG injection. First, a low‐dose CT scan (120 kV, 60–180 mA/s, slice thickness 3.75 mm) was performed. The PET scan was subsequently performed, with a whole‐body (including brain region) FDG‐PET scanning acquired for approximately 30–35 min. The brain imaging data were reconstructed into trans‐axial slices with a matrix size of 128 × 128 and a slice thickness of 3.3 mm.

After acquisition of PET images, all data were preprocessed by SPM12 software implanted in a MATLAB 2018a environment (MathWorks Inc.). The pre‐processing steps were as follows: first, the PET images were segmented and spatially normalized into a common Montreal Neurological Institute (MNI) atlas anatomical space following a 12‐parameter affine transformation and non‐linear transformations, yielding images composed of 2 mm × 2 mm × 2 mm voxels. Then, default SPM smoothing was applied using 12‐mm Gaussian kernel to increase the signal‐to‐noise ratio. For single subject analysis, the statistical basic models were performed between individual patient and controls using two‐sample t test model with age and gender as the nuisance variables. For groupwise analysis, FDG uptake was compared voxel‐by‐voxel between patients and controls group using a two‐sample *t*‐test of SPM. Significant results were viewed at the height threshold (*p* < 0.001) and corrected for multiple comparisons (familywise error [FWE] corrected or false discovery rate [FDR] corrected, *p* < 0.05). If significant clusters were not found, the more liberal threshold was considered (*p* < 0.001, uncorrected; extent threshold, *k* = 300).^17^

### 2.5 Follow‐up and prognosis analysis based on SPM

The follow‐up and clinical outcome information was obtained from outpatient visits and telephone interviews with patients or relatives. The modified Rankin Scale (mRS) was used to assess neurological disability at the last follow‐up in patients with anti‐GABAB receptor encephalitis^3^, ^18^, ^19^; patients were considered to have a good outcome if mRS score was ≤2 and poor outcome was defined as mRS score >2. In order to assess the potential imaging predictors on ^18^F‐FDG‐PET that might influence the long‐term outcome in subjects with anti‐GABAB receptor encephalitis, we performed group comparisons of ^18^F‐FDG‐PET data between patients with poor outcomes (*n* = 8) and good outcomes (*n* = 13) by two‐sample *t*‐test model of SPM12. The statistically significant and corrected method of multiple comparisons were same as aforementioned standards.

### 2.6 Statistical analysis

SPSS 22.0 software package for Windows (IBM Corp.) and Prism 7 (GraphPad software) were used for statistical analyses. The normality of data distribution of continuous variables was tested by one‐sample Shapiro‐Wilk test. Continuous variables with a normal distribution were presented as the mean ± standard deviation, and non‐normal variables were expressed as the median (interquartile range [IQR]). Continuous variables were compared using the *t*‐test or non‐parametric Mann–Whitney *U*‐test. Categorical variables were compared and analyzed by Fisher's exact test. With the exception of SPM analysis, a two‐tailed *p* value less than 0.05 (*p* < 0.05) was considered statistically significant.

## 3 RESULTS

### 3.1 Patient characteristics

The clinical details of 21 patients (15 male, 6 female) with anti‐GABAB receptor encephalitis are shown in Table 1. The median age of disease onset was 50 years (IQR, 46–64; range, 28–69 years), and seizures (21/21, 100%) were the most common clinical symptoms. In the population, 50% of patients showed more than one seizure types, and the main seizure classification was as follows: focal onset with awareness (14%), focal onset with impaired awareness (48%), generalized onset seizures (95%), and status epilepticus (SE, 14%). Other clinical presentations mainly included memory loss (*n* = 19, 90%), psychosis and abnormal behaviors (*n* = 13, 62%), sleep disturbances (*n* = 10, 48%), speech disorders (*n* = 2, 10%), and reduced level of consciousness (*n* = 2, 10%). Tumors were identified in 7 of 21 patients (33%). Both serum and CSF GABAB receptor antibody were observed in 16 patients (76%); the remaining 5 patients had GABAB receptor antibodies either in the serum (2/21, 10%) or in the CSF (3/21, 14%). All serum and CSF samples were negative for other neuronal cell‐surface antibodies (NMDAR, LGI1, CASPR2, AMPAR), whereas Hu antibodies were found in 3 patients with GABAB antibodies; other intracellular antibodies (Yo, Ri, Ma2, CV2, GAD65, and amphiphysin) were negative. The CSF pleocytosis was observed in 16 patients (76%), and protein concentration was elevated in 11 of 21 patients (52%). 71% of patients had positive oligoclonal bands. EEG features of all anti‐GABAB receptor encephalitis patients during the ictal and interictal phase were reviewed; a total of 16 patients (76%) experienced EEG abnormalities, which mainly included slow wave activities and epileptic discharges in the temporal regions.

**TABLE 1:** Patient characteristics (*n* = 21)

Clinical variables	Values
Age at onset, median (IQR, range), year	50 (46–64, 28–69)
Male, *n* (%)	15 (71%)
Clinical symptoms during disease course, *n* (%)	
Seizures	21 (100%)
Generalized seizures	20 (95%)
Focal seizures with impaired awareness	10 (48%)
Focal seizures without impaired awareness	3 (14%)
Status epilepticus	3 (14%)
Memory loss	19 (90%)
Psychosis and abnormal behaviors	13 (62%)
Sleep disturbances	10 (48%)
Speech disorders	2 (10%)
Reduced level of consciousness	2 (10%)
Tumors, *n* (%)	7 (33%)
GABAB receptor antibody, *n* (%)	21 (100%)
Both in serum and CSF	16 (76%)
Only in serum	2 (10%)
Only in CSF	3 (14%)
Associated paraneoplastic antibodies, *n* (%)	
Hu	3 (14%)
CSF abnormalities at onset, *n* (%)	
Pleocytosis	16 (76%)
Elevated protein levels	11 (52%)
Oligoclonal bands	15 (71%)
EEG abnormalities^a^, *n* (%)	16 (76%)
Initial MRI results, *n* (%)	
Normal	12 (57%)
MTL T2/FLAIR hyperintensities	9 (43%)
Unilateral	5 (24%)
Bilateral	4 (19%)
Treatment, *n* (%)	
Immunotherapy	21 (100%)
Tumor removal or chemotherapy	7 (33%)
Follow‐up and outcome	
Follow‐up time from onset, median (IQR), m	33 (16–52)
mRS at the last follow‐up, median (IQR)	2 (1–4)
Good outcome (mRS 0–2), *n* (%)	13 (62%)
Poor outcome (mRS 3–6), *n* (%)	8 (38%)

Abbreviations: CSF, cerebrospinal fluid; EEG, electroencephalogram; FLAIR, fluid‐attenuated inversion recovery; GABAB, gamma‐aminobutyric‐acid B; IQR, interquartile range; MRI, magnetic resonance imaging; mRS, modified Rankin Scale; MTL, medial temporal lobe.

^a^ Temporal area slow waves or epileptiform discharges were considered abnormal.

### 3.2 Comparisons of MRI and ^18^F‐FDG‐PET findings

**FIGURE 1:**
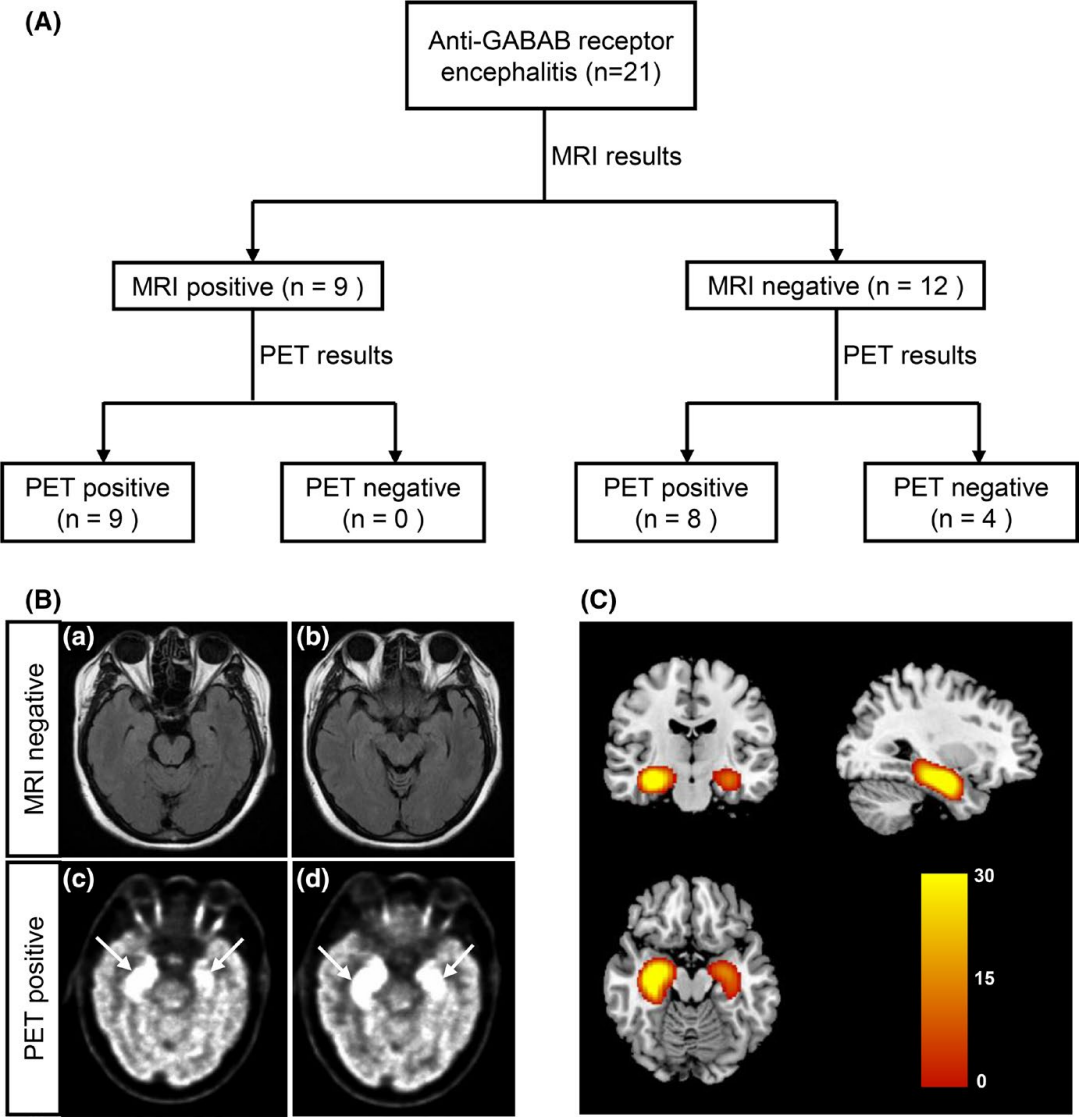
Comparisons between MRI and ^18^F‐FDG‐PET in patients with anti‐GABAB receptor encephalitis. (A) Neuroimaging testing plays an essential role in the diagnosis of anti‐GABAB receptor encephalitis. The sensitivity of ^18^F‐FDG‐PET is higher than that of MRI; (B) representative images in one patient with anti‐GABAB receptor encephalitis (patient #3): ^18^F‐FDG‐PET is positive (c and d) even if MRI is negative (a and b); (C) brain hypermetabolism limited to MTL structures in same representative case based on semi‐quantitative statistical parametric mapping method. Abbreviations: ^18^F‐FDG‐PET, ^18^F‐fluorodeoxy‐glucose positron emission tomography; GABAB, gamma‐aminobutyric‐acid B receptor; MTL, medial temporal lobe; MRI, magnetic resonance imaging

**FIGURE 2:**
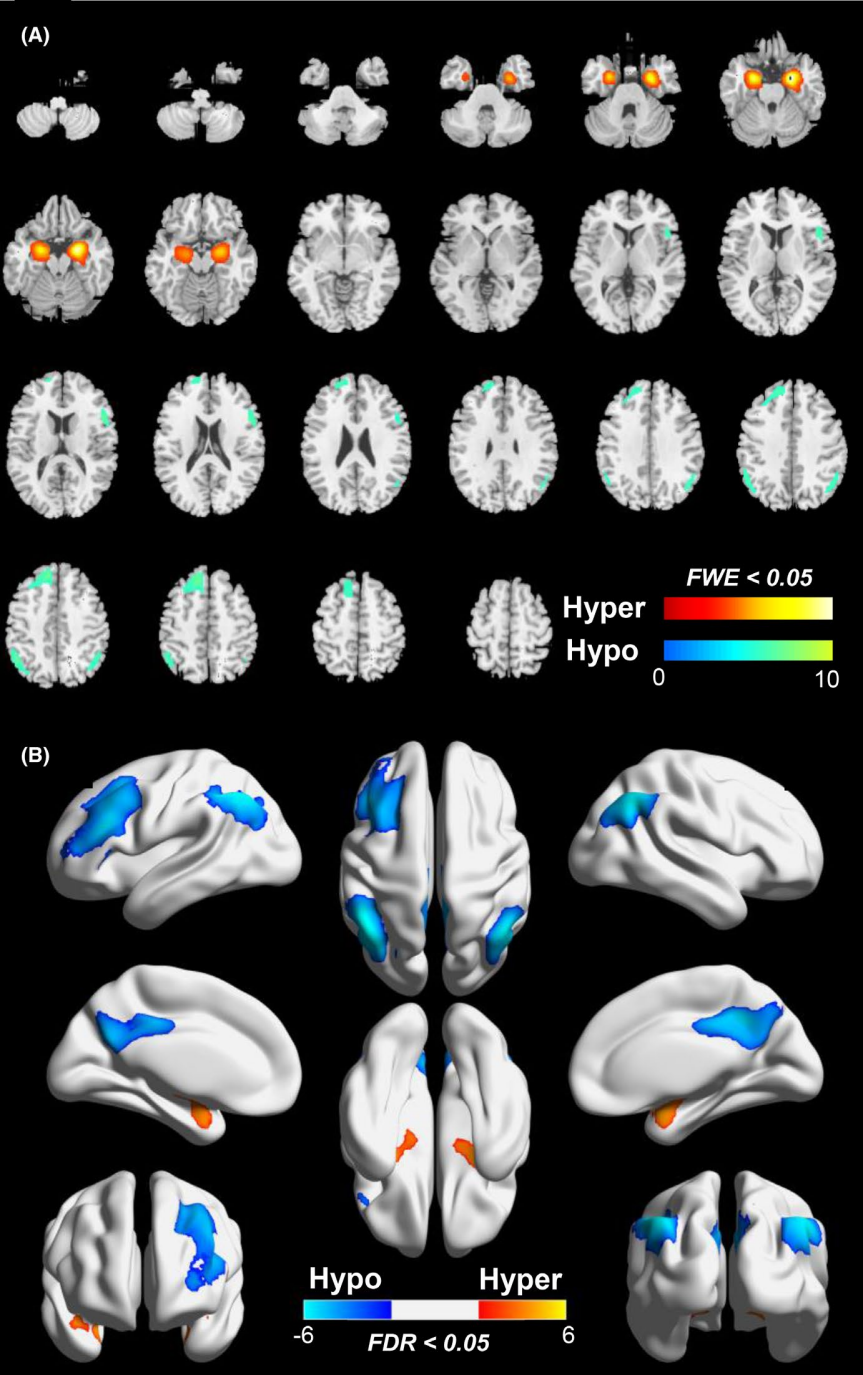
The results of ^18^F‐FDG‐PET pattern in anti‐GABAB receptor encephalitis. (A) Representative images of abnormal metabolism in individual patient (patient #13). Hypermetabolism pattern: MTL; hypometabolism pattern: frontal and parietal lobe (FWE corrected, *p* < 0.05). (B) Groupwise analysis confirms MTL hypermetabolism in association with relative hypometabolism in frontal and parietal lobe, extending to cingulate gyrus, is a general metabolic pattern in subjects with anti‐GABAB receptor encephalitis (FDR corrected, *p* < 0.05). Significant hyper‐ and hypometabolism is color coded as shown in legend. Renders created using Brain Net Viewer (Xia et al., 2013) (https://www.nitrc.org/projects/bnv/). Abbreviations: ^18^F‐FDG‐PET, ^18^F‐fluorodeoxy‐glucose positron emission tomography; FDR, false discovery rate; FEW, familywise error; GABAB, gamma‐aminobutyric‐acid B receptor; Hyper, hypermetabolism; Hypo, hypometabolism; MTL, medial temporal lobe

The MRI and ^18^F‐FDG‐PET findings in subjects with anti‐GABAB receptor encephalitis are summarized in Table 2. The brain MRIs were performed at a median of 34 days (IQR: 14–54 days) after disease onset and a median of 42 days (IQR: 34–63 days) from ^18^F‐FDG‐PET. There was no difference in the duration of symptoms to imaging between MRI and PET (*p* = 0.107). The cerebral MRI showed abnormal findings in 9 of 21 patients (42.9%), whereas abnormal metabolic patterns on ^18^F‐FDG‐PET were seen in 17 of 21 patients (81% vs. 42.9%, *p* = 0.025). Importantly, ^18^F‐FDG‐PET was 100% positive when MRI was positive, and ^18^F‐FDG‐PET was 67% positive even if there were no associated abnormalities on MRI (Figure 1A). For 12 patients with normal MRI, 5 patients (5/12, 41.7%) showed MTL hypermetabolism on ^18^F‐FDG‐PET (Table 2, Figure 1B, C). Of the 17 subjects with abnormal PET metabolism, the alter limbic lobe glucose metabolism (mostly hypermetabolism) was observed in 14 patients (14/21, 66.7%), of whom 10 (10/14, 71.4%) demonstrated hypermetabolism in the medial temporal lobe (MTL). ^18^F‐FDG‐PET demonstrated isolated cerebral hypermetabolism in 4 of 21 patients (19%), including brain regions in MTL, inferior temporal gyrus, basal ganglia, and cingulate gyrus. Isolated hypometabolism was noted in four patient (19%), which was related to cingulate gyrus and thalamus. Of the 21 patients, 9 (42.9%) revealed mixed metabolism patterns with hypermetabolism in combination with hypometabolism. Of those 9 patients, 7 (77.8%) with mixed metabolism mainly presented with a relatively common metabolic pattern consisting of MTL hypermetabolism and relative cortex hypometabolism (frontal and parietal lobe) (FWE corrected, *p* < 0.05; Figure 2A). In addition, groupwise analysis also confirmed MTL hypermetabolism in association with relative hypometabolism in frontal or parietal lobe, extending to cingulate gyrus, was a general metabolic pattern in subjects with anti‐GABAB receptor encephalitis (FDR corrected, *p* < 0.05; Figure 2B).

**TABLE 2:** Detailed MRI and ^18^F‐FDG‐PET features in patients with anti‐GABAB receptor encephalitis (*n* = 21)

Patient	Sex/Age	Time from onset to MRI (days)	MRI results	Time from onset to ^18^F‐FDG‐PET (days)	PET results of SPM analysis	
			T2/FLAIR hyperintensities		Hypermetabolism	Hypometabolism
1	M/62	31	Normal	35	MTL	Parietal lobe
2	F/69	54	MTL	35	Middle Temporal Gyrus, Parietal lobe	Frontal lobe
3	M/62	65	Normal	43	MTL	——
4	M/45	35	MTL	37	MTL	Parietal lobe
5	F/54	6	Normal	9	——	——
6	M/69	15	Normal	48	MTL	Frontal lobe, parietal lobe, transverse temporal gyrus, cingulate gyrus
7	F/32	3	Normal	25	——	Cingulate gyrus
8	M/49	12	Normal	39	MTL, Inferior temporal gyrus	——
9	F/50	29	Normal	42	——	——
10	M/28	27	MTL	65	——	Cingulate gyrus
11	M/50	68	MTL	50	MTL	Parietal lobe, cingulate gyrus
12	M/62	1	Normal	41	——	——
13	F/67	4	Normal	25	MTL	Frontal lobe, parietal lobe
14	M/44	35	Normal	25	——	Thalamus
15	M/47	80	MTL	70	MTL	——
16	M/45	86	MTL	112	Basal ganglia	Parietal lobe
17	M/47	74	MTL	76	——	Cingulate gyrus
18	M/57	53	Normal	68	——	——
19	M/65	23	MTL	46	MTL	Frontal lobe
20	M/69	45	MTL	61	MTL, Frontal lobe	Parietal lobe, inferior temporal gyrus
21	F/50	35	Normal	33	Basal ganglia, cingulate gyrus	——

Abbreviations: ^18^F‐FDG‐PET, ^18^F‐fluoro‐2‐deoxy‐d‐glucose positron emission tomography; F, female; FLAIR, fluid‐attenuated inversion recovery; GABAB, gamma‐aminobutyric‐acid B; M, male; MRI, magnetic resonance imaging; MTL, medial temporal lobe; SPM, statistical parametric mapping.

### 3.3 Correlation analysis between long‐term outcome and ^18^F‐FDG‐PET based on SPM

All patients received first‐line immunotherapy, including intravenous immunoglobulin and steroid pulse therapy, and 7 patients with tumors were additionally treated with chemotherapy or tumor removal (Table 1). After a median follow‐up of 33 months (IQR, 16–52 months) since symptom onset, 13 of 21 patients (62%) showed a good outcome, whereas a poor outcome was observed in 8 patients (38%, Table 1). Three patients (14.3%) died of cancer malignancy progression. The clinical relapses in anti‐GABAB receptor encephalitis were not common; only 1 patient developed recurrence after 46 months from onset.

**FIGURE 3:**
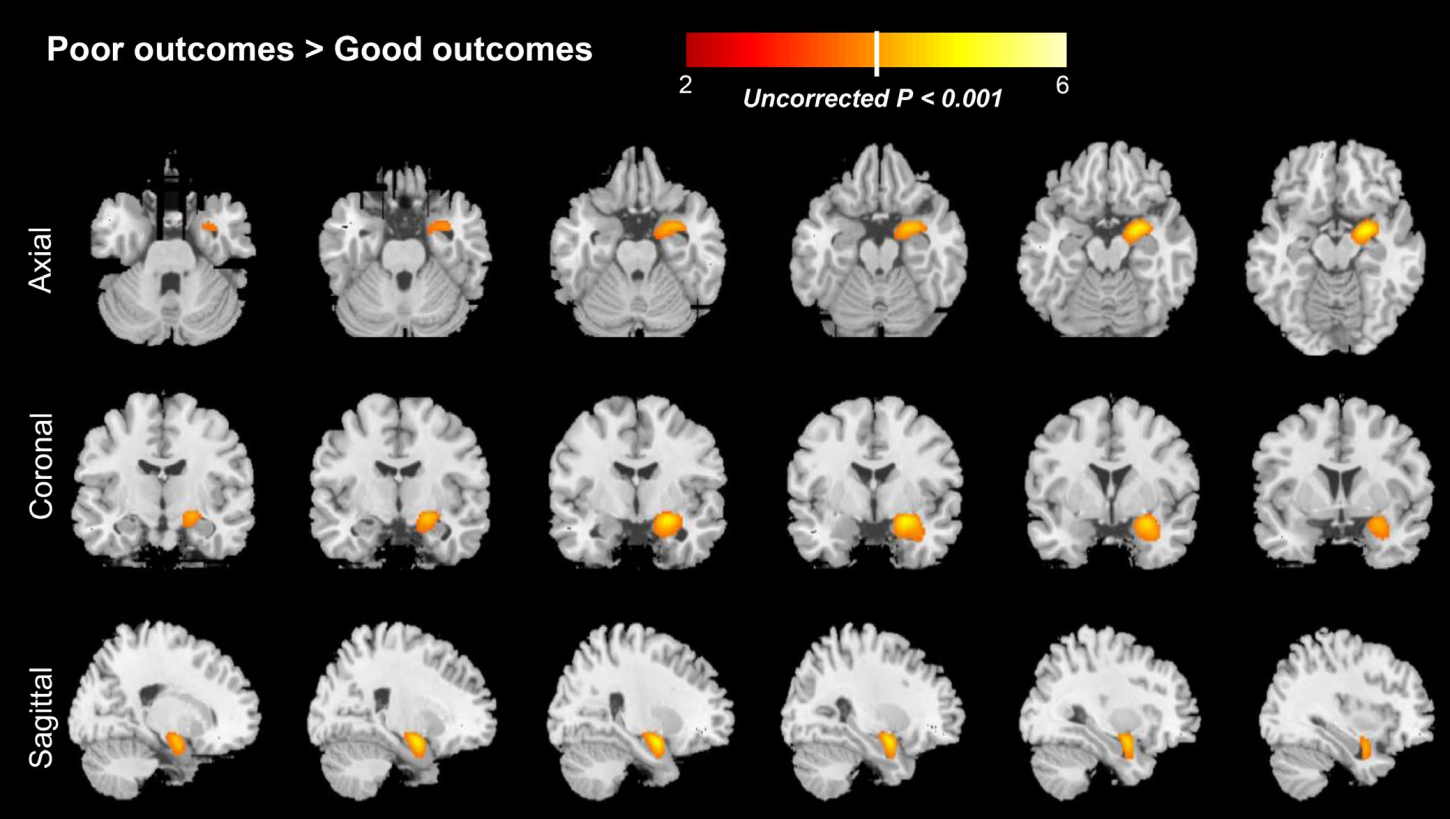
Correlation analysis between long‐term outcome and ^18^F‐FDG‐PET based on statistical parametric mapping method in patients with anti‐GABAB receptor encephalitis. Compared to patients with good outcome, patients with poor outcome demonstrated relatively increased metabolism in the MTL (uncorrected, *p* < 0.001). Abbreviations: ^18^F‐FDG‐PET, ^18^F‐fluorodeoxy‐glucose positron emission tomography; GABAB, gamma‐aminobutyric‐acid B receptor; MTL, medial temporal lobe

The baseline comparison between patients with good and poor outcomes is shown in Table 3. Tumors were more frequently observed in patients with poor outcomes than those with good outcomes (*p* = 0.003). However, there were no other differences between patients with good outcomes (*n* = 13) and poor outcomes (*n* = 8), including age, sex, specific clinical features (seizures, cognitive deficits, psychosis and change of behaviors, sleep disorders), CSF findings, EEG, and MRI, treatments, although the power was limited due to the sample size. In addition, the median interval from symptoms onset to PET between prognostic subgroups did not reach statistical significance (*p* = 0.203). Further group comparisons of ^18^F‐FDG‐PET pattern based on voxel‐based analysis of SPM showed patients with poor outcomes demonstrated relatively increased metabolism in the MTL compared to those with good outcomes in anti‐GABAB receptor encephalitis (uncorrected, *p* < 0.001, Figure 3).

**TABLE 3:** Baseline comparison between patients with good outcomes and poor outcomes

	Patients with good outcomes (mRS 0–2, *n* = 13)	Patients with poor outcome (mRS 3–6, *n* = 8)	Comparison (*p* Value)
Median age (IQR), year	50 (46–60)	64 (46–69)	0.215
Sex, *n* (%)			
Male	8 (62%)	7 (88%)	0.336
Main symptoms of encephalitis, *n* (%)			
Seizures	13 (100%)	8 (100%)	>0.05
Cognitive deficits	11 (85%)	8 (100%)	0.505
Psychosis and change of behaviors	6 (46%)	7 (88%)	0.085
Sleep disorders	4 (31%)	6 (75%)	0.080
Tumors, *n* (%)	1 (8%)	6 (75%)	0.003*
CSF findings, *n* (%)			
Pleocytosis	8 (62%)	8 (100%)	0.111
Elevated protein levels	5 (38%)	6 (75%)	0.183
EEG abnormalities^a^, *n* (%)	9 (69%)	7 (88%)	0.606
MRI results, *n* (%)			
MTL T2/FLAIR hyperintensities	4 (31%)	5 (63%)	0.203
Immunotherapy, *n* (%)	13 (100%)	8 (100%)	>0.05
Median interval between symptoms onset and ^18^F‐FDG‐PET, IQR, days	39 (29–58)	47 (39–68)	0.203

Abbreviations: ^18^F‐FDG‐PET, ^18^F‐fluoro‐2‐deoxy‐d‐glucose positron emission tomography; CSF, cerebrospinal fluid; EEG, electroencephalogram; FLAIR, fluid‐attenuated inversion recovery; IQR, interquartile range; MRI, magnetic resonance imaging; mRS, modified Rankin Scale; MTL, medial temporal lobe.

^a^ Temporal area slow waves or epileptiform discharges were considered abnormal.

* *p* < 0.05.

## 4 DISCUSSION

Our study has three major highlights and clinical implications. First, we revealed highly pronounced MTL hypermetabolism in association with relative frontal and parietal hypometabolism detected by semi‐quantitative brain ^18^F‐FDG‐PET in patients with anti‐GABAB receptor encephalitis compared to controls. Second, this study also confirmed that ^18^F‐FDG‐PET may be relatively sensitive in the early diagnosis of anti‐GABAB receptor encephalitis, especially in the absence of any abnormal MRI findings; thus, ^18^F‐FDG‐PET should be considered when patients with normal MRI for suspected anti‐GABAB receptor encephalitis; Third, we also found that patients with poor outcomes had more significant hypermetabolism in the MTL compared with controls and patients with good outcomes, suggesting that pronounced MTL hypermetabolism may be an imaging biomarker of poor prognosis in patients with anti‐GABAB receptor encephalitis. Overall, the clinical application of ^18^F‐FDG‐PET may not only help in early diagnosing anti‐GABAB receptor encephalitis, but also in predicting long‐term outcomes.

Similar to prior reports, the majority of anti‐GABAB receptor encephalitis patients presented with symptoms of limbic encephalitis, such as seizures, memory loss, and mental and behavioral disorders.^4^, ^15^ Additionally, around two thirds of patients with anti‐GABAB receptor encephalitis indicated abnormal metabolism located in the limbic lobe (mostly in MTL) on ^18^F‐FDG‐PET in our study. Thus, these clinical and imaging findings hint toward predominant involvement of the limbic system in anti‐GABAB receptor encephalitis. In addition to metabolic lesions in MTL, our study also showed functional metabolic changes on FDG‐PET in the extra‐limbic structures, including basal ganglia, thalamus, frontal, temporal, and parietal lobe cortex.^1^, ^2^, ^20^ MTL hypermetabolism was the most prominent PET feature of anti‐GABAB receptor encephalitis as previously described,^11^ and only approximately 10% patients showed involvement of basal ganglia. In encephalitis associated with LGI1 antibody, MTL and basal ganglia hypermetabolism have been systematically described.^16^, ^21^ In addition, patients with anti‐NMDAR encephalitis usually show a relative hypermetabolism in the frontal, temporal lobe and basal ganglia, hypometabolism in the parietal and occipital lobe.^22^, ^23^, ^24^, ^25^ Hence, compared with anti‐LGI1 and anti‐NMDAR encephalitis, patients with anti‐GABAB receptor encephalitis usually have limited involvement of the basal ganglia, which suggests that, in addition to whole‐body tumor screening, ^18^F‐FDG‐PET may also be helpful in differentiating antibodies subtypes of AE. However, the sample size is relatively small and the pathophysiological mechanism of this metabolic signature, which might be related to the distribution of GABAB or other receptors in the brain, is still unclear. Recently, many studies have shown some specific PET tracers, such as [18F] cEFQ and [18F]GE‐179, that have been developed for mapping excitatory receptor activations in the brain.^26^, ^27^ These tracers could potentially be used to map and localize regional differences in abnormal receptor distribution in the brain using PET and to investigate pathophysiological basis of different metabolic features. Future studies should focus on novel and specific PET tracers for receptors associated with AE and further validate the clinical value of receptor activations in the mechanism of AE.

The early diagnosis of AE is still challenging due to the fact that patients manifest with various unspecific symptoms, and there is often a delay or unavailability of antibody detection. A recent clinical diagnosis criterion affirms the potential role of brain imaging in the diagnosis and evaluation for AE,^5^, ^7^ but still mainly relies on MRI. ^18^F‐FDG‐PET is only incorporated into diagnostic criteria for definite autoimmune limbic encephalitis, which may result from the uncertainty of its positive and negative predictive value for the diagnosis of various AE subtypes.^28^ Although there have been many prior studies of ^18^F‐FDG‐PET in autoimmune encephalitis,^29^ the FDG‐PET pattern in anti‐GABAB receptor encephalitis is still uncertain. As an effective supplement, our cohort showed that ^18^F‐FDG‐PET might be more sensitive in the early diagnosis than MRI in patients with anti‐GABAB receptor encephalitis. Hence, a standardized ^18^F‐FDG‐PET approach is needed to meet the requirements for diagnosing anti‐GABAB receptor encephalitis.

Generally, early immunotherapy facilitates favorable outcomes for anti‐GABAB receptor encephalitis^3^; however, the prognostic predictors of anti‐GABAB receptor encephalitis remains unknown. Previous case studies have demonstrated that poor prognosis might be relevant with status epilepticus, respiratory failure, and malignant tumors.^4^, ^30^ Moreover, recent studies have shown that the involvement of limbic system on MRI was more common in the poor prognosis group than in the favorable prognosis group.^31^ In our study, altered glucose metabolism in the MTL observed by ^18^F‐FDG‐PET was related to poor clinical outcomes in patients with anti‐GABAB receptor encephalitis. Thus, regardless of MRI or FDG‐PET, we conclude that involvement of limbic structures is associated with a poor prognosis in patients with anti‐GABAB receptor encephalitis. Future prospective studies are required to verify these findings and clarify potential pathogenic mechanisms.

There are several limitations in this study. First, a potential selection bias exists based on the retrospective nature of this study. Second, the sample size may be relatively small in this study due to low prevalence of anti‐GABAB receptor encephalitis and high economic burden of ^18^F‐FDG‐PET examinations. In addition, ^18^F‐FDG‐PET was not performed in all subjects diagnosed with anti‐GABAB receptor encephalitis, which may equally result in small sample size. Thus, these findings are exploratory and the PET results in this small cohort should be replicated in future trials with larger sample sizes. Third, ^18^F‐FDG‐PET in all included patients were only performed in acute phase of disease, and none patients underwent ^18^F‐FDG‐PET after treatment or at follow‐up, and thus, we may fail to evaluate the role in the aspect of treatment effect, further prospective and longitudinal cohort studies should be performed.

## 5 CONCLUSIONS

In summary, this study demonstrates a previously unidentified role of ^18^F‐FDG‐PET imaging in the diagnosis and outcome of anti‐GABAB receptor encephalitis. Acute ^18^F‐FDG‐PET was more sensitive than MRI in the early diagnosis of anti‐GABAB receptor encephalitis. A general metabolic pattern in anti‐GABAB receptor encephalitis is MTL hypermetabolism in association with relative frontal or parietal hypometabolism, suggesting that MTL hypermetabolism may serve as a prognostic biomarker in anti‐GABAB receptor encephalitis. Future larger prospective studies are needed to further clarify the role of ^18^F‐FDG‐PET in the diagnosis and management for various subtypes of AE.

## CONFLICT OF INTEREST

The authors declare that they have no competing interests.

## AUTHOR CONTRIBUTIONS

XL and QW recruited, diagnosed, and assessed patients. XL, TY, XZ, GL, RL, LA, and QW worked on the establishment of the separate databases. XL drafted a significant portion of the manuscript or figures. LA and QW re‐analyzed and interpreted all final data. All authors contributed to the current version of the article either regarding conception or design, data analysis, or editing, and all authors read and approved the final manuscript.

## Data Availability

The data are not publicly available due to privacy or ethical restrictions. The data that support the findings of this study are available on request from the corresponding author.
